# Association between dietary copper intake and bone mineral density in children and adolescents aged 8–19 years: A cross-sectional study

**DOI:** 10.1371/journal.pone.0310911

**Published:** 2024-10-01

**Authors:** Aiyong Cui, Juan Yan, Haoran Li, Zhiqiang Fan, Xing Wei, Hu Wang, Yan Zhuang

**Affiliations:** 1 Department of Orthopaedics, Honghui Hospital, Xi’an Jiao Tong University, Xi’an, China; 2 Department of Medical Services Section, The Seventh Affiliated Hospital of Sun Yat-sen University, Shen’zhen, China; Gent University, BELGIUM

## Abstract

**Purpose:**

Some studies showed the possible role of copper intake on bone mineral density (BMD) in adults or the elderly, but the association remained uncertain in children and adolescents. Our research explored the association between copper intake and BMD in individuals aged 8–19 years from the National Health and Nutrition Examination Survey (NHANES) 2011–2016.

**Methods:**

In the present study, 6,965 individuals aged 8–19 (mean age 13.18 ± 3.38 years) were enrolled from the NHANES 2011–2016. Copper intake was evaluated by averaging two 24-hour copper dietary intake recalls. Multivariate linear regression analyses were used to explore the association between copper intake and total BMD, subtotal BMD, and total spine BMD in children and adolescents. Stratified analyses and interaction tests were performed by age, gender, and race.

**Results:**

Participants of the higher quartile of copper intake were more likely to be older, men, Non-Hispanic White, and Other Hispanic. They have higher values of poverty income ratio (PIR), serum phosphorus, blood urea nitrogen, serum vitamin D, and BMD and lower values of body mass index (BMI), cholesterol, total protein, and serum cotinine. In the fully adjusted model, we found positive associations between copper intake and total BMD (β = 0.013, 95CI: 0.006, 0.019)), subtotal BMD (β = 0.020, 95CI: 0.015, 0.024), and total spine BMD (β = 0.014, 95CI: 0.009, 0.019). Stratified analyses showed that the association was stronger in men, individuals aged 14–19, Non-Hispanic White, and Other Hispanic.

**Conclusions:**

Our study suggests that copper intake is positively associated with BMD in U.S. children and adolescents. The study emphasizes the role of copper intake on bone health in the early stages of life. However, more investigations are needed to verify our findings and their underlying mechanisms.

## Introduction

Osteoporosis is a common skeletal disease that threatens one-fifth of older people around the world [1]. In the U.S., it is estimated that 43.4 million people had low bone mass, and 10.2 million had osteoporosis in 2010, placing a massive burden on society [2]. Based on recent evidence, osteoporosis can be ascribed to various factors, including aging, sex, genetics, environment, nutrition, and others [3,4].

Trace elements in a daily diet are considered important factors influencing bone health [5,6]. Copper is an essential trace element for human health, which can only be obtained from dietary sources [7]. Evidence indicates that copper is key in maintaining normal body metabolism, including the brain, cardiovascular system, and other functions [8]. Furthermore, evidence has suggested that copper may play a positive role in maintaining bone health [9–11]. In an early study by Chaudhri et al. [9], they found that plasma copper can be a simple and sensitive indicator of bone health in postmenopausal women. In a National Health and Nutrition Examination Survey (NHANES) study, Qu et al. [10] found that moderate serum copper levels are essential for bone health, and higher serum copper levels were significantly associated with fracture incidence in men. However, the study’s small sample (722 subjects) may limit the interpretation of this result. In a recent study by Fan et al. [12], a large sample of 8224 individuals from NHANES were enrolled in the study. They found that individuals in the highest quartile of copper intake had a higher BMD and lower risk of osteoporosis compared with the first quartile.

To our knowledge, no study has explored the association between copper intake and BMD in children and adolescents. Previous evidence suggested that most bone mass accumulation occurs in childhood and adolescence [13]. Thus, maximizing peak bone mass (PBM) in adolescence is an important measure to prevent fragility fractures and osteoporosis in older age [14]. Studies have shown that a 10% increase in PBM during adolescence could decrease 50% fracture risk in older age [15]. Our study aims to assess the association between copper intake and BMD in subjects aged 8–19 using NHANES 2011–2016 data, aiming to provide crucial information on bone health during the early stages of life.

## Methods

### Study design

**Fig 1:**
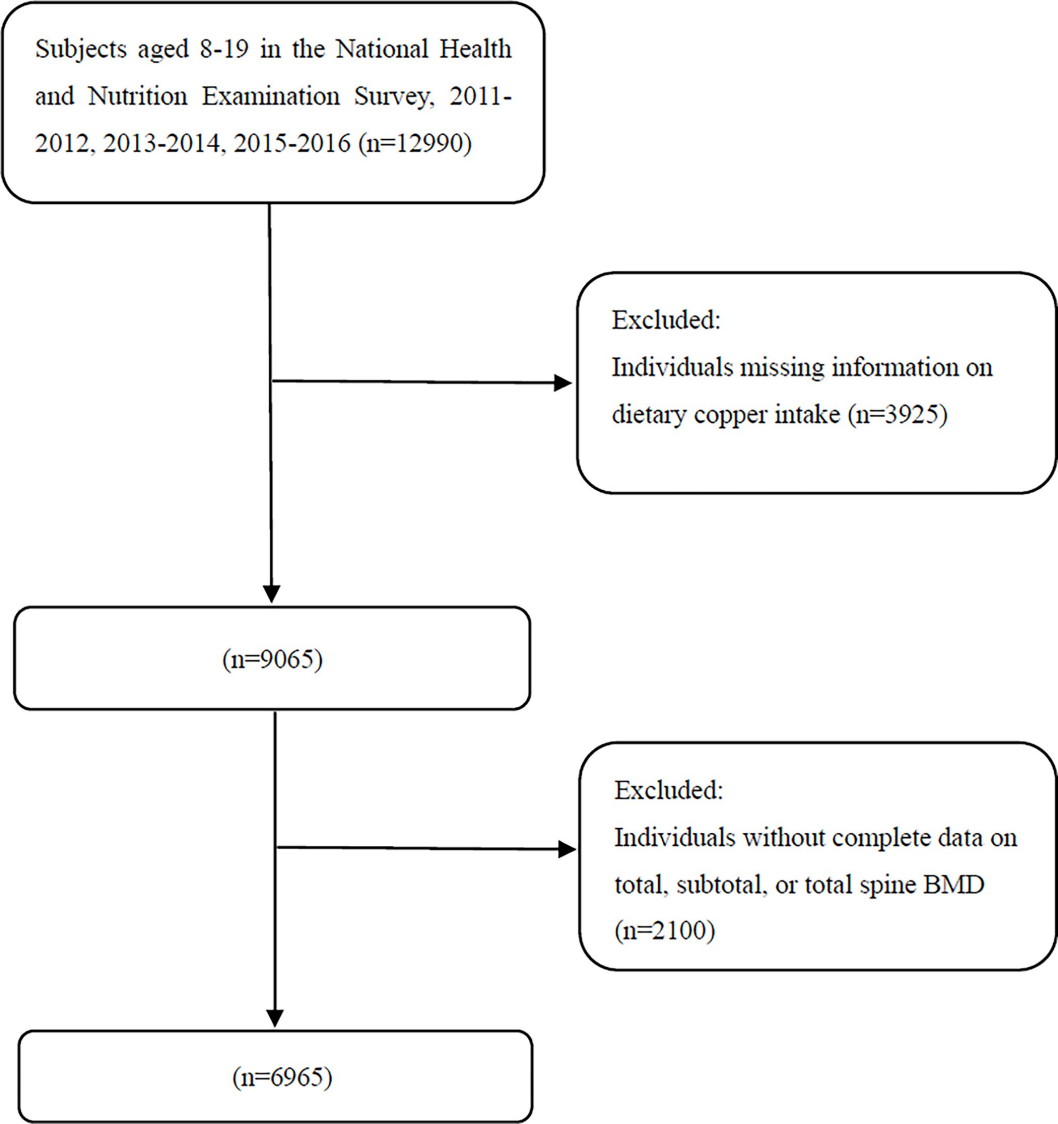
Flowchart of participants selection. BMD, bone mineral density.

NHANES is a national nutrition program conducted by Disease Control and Prevention, which uses a multistage complex and probability design. The National Center for Health Statistics (NCHS) Research Ethics Review Board approved the project. All subjects or their guardians signed informed consent forms for the programs. We combined three cycles from the NHANES 2011–2012, 2013–2014 and 2015–2016. Initially, 12990 subjects aged 8–19 years were enrolled from 2011–2016. Then, participants were excluded because of lacking information on dietary copper (N = 3925) or BMD data (N = 2100); 6,965 participants were ultimately enrolled for analysis (S1 Appendix). The detailed process of participant selection is shown in Fig 1. The study does not need any ethics approval because it incorporates and synthesize data from public database.

### Variables

#### Dietary copper intake

Dietary copper intake was assessed by two 24-hour dietary recalls to reduce the recall bias. The first dietary recall interview was performed at the Mobile Examination Center (MEC), followed by a second interview 3 to 10 days later via a return phone call. Each dietary copper intake was evaluated by all foods and beverages intake according to the Food and Nutrient Database for Dietary Studies of the United States Department of Agriculture [16]. The final copper intake was estimated by averaging two 24-hour copper dietary intake recalls.

#### Measurement of BMD

The variables included total BMD, subtotal BMD, and total spine BMD. Trained and certified radiology technologists measured BMD by dual-energy X-ray absorptiometry (DXA) (Hologic, Inc., Bedford, Massachusetts). Then, the scan results were analyzed using Hologic APEX v3.0 software. All BMD data were collected and standardized for further analysis. The subjects were tested using a single BMD device for all subtypes. Detailed information on BMD data can be seen in DXDTOBMD, DXDSTBMD, and DXXLSBMD datasets on the NHANES website [17]. The Body Composition Procedures Manual on the NHANES website describes the DXA examination protocol (CDC Dual Energy X-Ray Absorptiometry—Whole Body) [18].

#### Covariates

The covariates were included to eliminate potential effects on the overall results, according to the published studies [19,20]: age, gender, race, poverty income ratio (PIR), body mass index (BMI), serum vitamin D, serum total protein, serum calcium, serum phosphorus, blood urea nitrogen, cholesterol, serum uric acid, serum cotinine, and physical activity. PIR was calculated by dividing family income by the poverty threshold in the survey year [21]. Physical activity was measured by questionnaires: During the past 7 days, on how many days did you get at least 60 minutes of physical activity? Detailed information on demographics, laboratory data, questionnaires, and additional covariates can be found on the NHANES website (https://www.cdc.gov/nchs/nhanes/).

### Statistical analysis

Mean ± standard deviation and percentages were used to represent continuous and categorical variables. We employed weighted linear regression models and weighted χ2 tests to compare differences between continuous and categorical variables. Multivariate logistic regression analyses were used to evaluate the association of copper intake with total BMD, subtotal BMD, and total spine BMD. Then, we changed the continuous variable of cooper intake into a categorical variable (quartiles). Multivariate logistic regression analyses were used to explore the association between copper intake (quartiles) and BMD in children and adolescents. Trend tests were used to analyze their linear association trend. We built three models: Model 1 (unadjusted), Model 2 (age, race, and sex), Model 3 (age, gender, race, PIR, BMI, serum vitamin D, serum total protein, serum calcium, serum phosphorus, blood urea nitrogen, cholesterol, serum uric acid, serum cotinine, and physical activity). Stratified analyses and interaction tests were performed by sex (male and female), age (8–13 and 14–19), and race/ethnicity (Mexican American, Non-Hispanic White, Other Hispanic, Non-Hispanic Black, and Other Race). All analyses were performed by R software (4.3.2) and EmpowerStats (4.0), using complex MEC weighted to ensure population representation. P values less than 0.05 were regarded as statistically significant.

## Results

The baseline characteristics of participants were analyzed according to copper intake (Q1-Q4), as listed in Table 1. In total, 6,965 subjects aged 8–19 years were enrolled in our study, of which 52.19% of subjects were men and 47.81% were women. The mean age of participants was 13.18 ± 3.38 years. Moreover, 22.99% of the participants were Mexican American, 27.60% were non-Hispanic white, 22.79% were non-Hispanic black, 14.83% were other races (including multiracial), and 11.80% were other Hispanic. Participants’ average total BMD, subtotal BMD, and total spine BMD were 0.94 ± 0.16, 0.84 ± 0.16, and 0.87 ± 0.19 g/cm^2^. Compared with those in the lowest quartile of copper intake, participants of the highest quartile of copper intake were more likely to be older, men, Non-Hispanic White, and Other Race. They engage in more physical activities and have higher values of PIR, serum phosphorus, blood urea nitrogen, and serum vitamin D, as well as total, subtotal, and total spine BMD. Furthermore, they have lower BMI, cholesterol, total protein, and serum cotinine values.

**Table 1:** Characteristics of the study population based on dietary copper intake quartiles.

		Copper intake quartiles				
	Total	Q1(<0.586)	Q2 (0.586–0.831)	Q3(0.831–1.138)	Q4(>1.138)	P value
Number of subjects (n)	6965	1740	1742	1740	1743	
Age (years)	13.18 ± 3.38	13.30 ± 3.37	12.76 ± 3.34	13.18 ± 3.38	13.50 ± 3.41	<0.001
Gender (%)						<0.001
Men	3635 (52.19%)	761 (43.74%)	875 (50.23%)	863 (49.60%)	1136 (65.17%)	
Women	3330 (47.81%)	979 (56.26%)	867 (49.77%)	877 (50.40%)	607 (34.83%)	
Race/ethnicity (%)						<0.001
Mexican American	1601 (22.99%)	422 (24.25%)	406 (23.31%)	407 (23.39%)	366 (21.00%)	
Other Hispanic	822 (11.80%)	232 (13.33%)	174 (9.99%)	200 (11.49%)	216 (12.39%)	
Non-Hispanic White	1922 (27.60%)	397 (22.82%)	494 (28.36%)	544 (31.26%)	487 (27.94%)	
Non-Hispanic Black	1587 (22.79%)	512 (29.43%)	450 (25.83%)	313 (17.99%)	312 (17.90%)	
Other Race	1033 (14.83%)	177 (10.17%)	218 (12.51%)	276 (15.86%)	362 (20.77%)	
BMI	22.64 ± 6.18	23.67 ± 6.67	22.56 ± 6.31	22.13 ± 5.80	22.19 ± 5.76	<0.001
PIR	2.02 ± 1.49	1.69 ± 1.30	1.94 ± 1.41	2.13 ± 1.55	2.31 ± 1.60	<0.001
Serum total calcium (mmol/L)	9.57 ± 0.23	9.57 ± 0.22	9.57 ± 0.24	9.56 ± 0.22	9.57 ± 0.22	0.205
Serum phosphorus (mmol/L)	4.35 ± 0.51	4.31 ± 0.49	4.37 ± 0.51	4.37 ± 0.51	4.37 ± 0.51	<0.001
Blood urea nitrogen	11.44 ± 2.56	11.17 ± 2.58	11.31 ± 2.41	11.46 ± 2.65	11.83 ± 2.53	<0.001
Cholesterol	157.86 ± 22.22	159.60 ± 23.26	157.55 ± 20.95	157.75 ± 20.87	156.55 ± 23.56	0.005
Total protein	7.28 ± 0.31	7.31 ± 0.31	7.28 ± 0.29	7.27 ± 0.31	7.27 ± 0.31	<0.001
Vitamin D	57.95 ± 18.49	54.69 ± 16.27	58.05 ± 18.27	59.18 ± 20.44	59.85 ± 18.34	<0.001
Cotinine	5.07 ± 31.79	6.95 ± 33.71	5.65 ± 41.33	2.93 ± 19.35	4.76 ± 28.59	<0.001
Exercise days of at least 60 minutes in one typically week						<0.001
≤3	1573 (22.58%)	473 (27.18%)	413 (23.71%)	394 (22.64%)	293 (16.81%)	
≥4	3280 (47.09%)	725 (41.67%)	887 (50.92%)	824 (47.36%)	844 (48.42%)	
No reported	2112 (30.32%)	542 (31.15%)	442 (25.37%)	522 (30.00%)	606 (34.77%)	
Total BMD (g/cm2)	0.94 ± 0.16	0.95 ± 0.16	0.93 ± 0.16	0.94 ± 0.16	0.96 ± 0.16	<0.001
Subtotal femur BMD (g/cm2)	0.84 ± 0.16	0.84 ± 0.15	0.82 ± 0.16	0.83 ± 0.16	0.86 ± 0.16	<0.001
Total spine BMD (g/cm2)	0.87 ± 0.19	0.88 ± 0.19	0.85 ± 0.20	0.86 ± 0.19	0.88 ± 0.19	<0.001
Copper intake (mg/d)	0.93 ± 0.43	0.51 ± 0.11	0.76 ± 0.06	0.98 ± 0.07	1.48 ± 0.45	<0.001

Mean ± SD for continuous variables: The P value was calculated by the weighted linear regression model. (%) for categorical variables: The *P* value was calculated by the weighted chi-square test. Abbreviation: BMD, bone mineral density. BMI, body mass index. PIR, poverty income ratio.

### Association between dietary copper intake and BMD

Table 2 displays the association between copper intake and BMD. In the unadjusted model, we found that copper intake was positively associated with subtotal BMD (β = 0.025, 95CI: 0.017, 0.034) and total spine BMD (β = 0.019, 95CI: 0.010, 0.028), but not total BMD (β = 0.007, 95CI: -0.003, 0.018). In model 2, multivariate linear regression analyses showed a positive association between copper intake and total BMD (β = 0.007, 95CI: 0.001, 0.013), subtotal BMD (β = 0.014, 95CI: 0.008, 0.019), and total spine BMD (β = 0.009, 95CI: 0.003, 0.014) in children and adolescents. In the fully adjusted model, we found positive associations between copper intake and total BMD (β = 0.013, 95CI: 0.006, 0.019), subtotal BMD (β = 0.020, 95CI: 0.015, 0.024), and total spine BMD (β = 0.014, 95CI: 0.009, 0.019). Then, we convert continuous variables (dietary copper intake) to categorical variables (quartiles). In the fully adjusted model, compared with people with the lowest quartile of copper intake, subjects in the highest quartile of copper intake had an increased BMD at total BMD (β = 0.014, 95CI: 0.008, 0.020), subtotal BMD (β = 0.020, 95CI: 0.014, 0.025), and total spine (β = 0.017, 95CI: 0.009, 0.024).

**Table 2:** The association between dietary copper intake and BMD.

		Model 1		Model 2		Model 3	
		β (95% CI)	P value	β (95% CI)	P value	β (95% CI)	P value
Total BMD		0.007 (-0.003, 0.018)	0.182	0.007 (0.001, 0.013)	0.033	0.013 (0.006, 0.019)	<0.001
	Q1	Reference		Reference		Reference	
	Q2	0.022 (-0.033, -0.012)	<0.001	-0.001 (-0.007, 0.005)	0.771	0.002 (-0.004, 0.008)	0.426
	Q3	-0.013 (-0.024, -0.003)	0.013	-0.000 (-0.007, 0.006)	0.879	0.004 (-0.002, 0.010)	0.205
	Q4	0.010 (-0.001, 0.020)	0.07	0.009 (0.002, 0.015)	0.008	0.014 (0.008, 0.020)	<0.001
	P for trend	0.024		0.011		<0.001	
Subtotal BMD		0.025 (0.017, 0.034)	<0.001	0.014 (0.008, 0.019)	0.001	0.020 (0.015, 0.024)	<0.001
	Q1	Reference		Reference		Reference	
	Q2	-0.018 (-0.029, -0.008)	<0.001	0.002 (-0.004, 0.008)	0.526	0.006 (0.000, 0.012)	0.043
	Q3	-0.010 (-0.021, 0.000)	0.051	0.001 (-0.005, 0.007)	0.706	0.006 (0.000, 0.012)	0.034
	Q4	0.017 (0.007, 0.028)	0.001	0.014 (0.007, 0.020)	<0.001	0.020 (0.014, 0.025)	<0.001
	P for trend	<0.001		<0.001		<0.001	
Total spine BMD		0.019 (0.010, 0.028)	<0.001	0.009 (0.003, 0.014)	0.001	0.014 (0.009, 0.019)	<0.001
	Q1	Reference		Reference		Reference	
	Q2	-0.031 (-0.044, -0.018)	<0.001	-0.002 (-0.009, 0.006)	0.679	0.003 (-0.005, 0.011)	0.438
	Q3	-0.021 (-0.034, -0.009)	0.001	-0.003 (-0.011, 0.004)	0.373	0.001 (-0.007, 0.008)	0.882
	Q4	-0.003 (-0.016, 0.009)	0.613	0.009 (0.001, 0.017)	0.019	0.017 (0.009, 0.024)	<0.001
	P for trend	0.998		0.041		<0.001	

Model 1: No covariates were adjusted. Model 2: Age, gender, and race were adjusted. Model 3: Age, gender, race, PIR, BMI, serum vitamin D, serum total protein, serum calcium, serum phosphorus, blood urea nitrogen, cholesterol, serum uric acid, serum cotinine, and physical activity.

Abbreviation: BMD, bone mineral density. PIR, poverty income ratio. BMI, body mass index.

### Stratification analysis

Stratified analyses were conducted by age, sex, and race (Table 3). In the fully adjusted model, we found that positive association was significant in men but not women (P for interaction< 0.05). For men, every unit increase in copper intake could improve total BMD, subtotal BMD, and total spine BMD by 0.016 g/cm^2^ (β = 0.016, 95CI: 0.010–0.022), 0.017 g/cm^2^ (β = 0.017, 95CI: 0.011–0.023), and 0.015 g/cm^2^(β = 0.015, 95CI: 0.007–0.022). Stratified analyses by age group showed that the positive association was more significant in those ages 14–19 years than in 8–13 years (P for interaction< 0.05). Every unit increase in copper intake is associated with 0.015 g/cm^2^ higher total BMD (β = 0.015, 95CI: 0.007–0.024), 0.019 g/cm^2^ higher subtotal BMD (β = 0.019, 95CI: 0.011–0.026), and 0.013 g/cm^2^ higher total spine BMD (β = 0.013, 95CI: 0.002–0.023) in individuals aged 14–19, respectively. Furthermore, stratified analyses by race suggested that the positive association was more prominent in non-Hispanic whites and Other Hispanics (P for interaction< 0.05) (Table 3).

**Table 3:** Association between dietary copper intake and BMD for stratified analyses.

	Total BMD			Subtotal BMD			Total spine BMD		
	Model1 β (95% CI) P value	Model2 β (95% CI) P value	Model3 β (95% CI) P value	Model1 β (95% CI) P value	Model2 β (95% CI) P value	Model3 β (95% CI) P value	Model1 β (95% CI) P value	Model2 β (95% CI) P value	Model3 β (95% CI) P value
Stratified by sex									
Male	**0.041 (0.030, 0.052) <0.001**	**0.010 (0.003, 0.016) 0.003**	**0.016 (0.010, 0.022) <0.001**	**0.043 (0.031, 0.054) <0.001**	**0.011 (0.004, 0.017) 0.001**	**0.017 (0.011, 0.023) <0.001**	**0.050 (0.037, 0.062) <0.001**	**0.011 (0.003, 0.018) 0.006**	**0.015 (0.007, 0.022) <0.001**
Female	**-0.036 (-0.051, -0.020) <0.001**	-0.000 (-0.009, 0.009) 0.943	0.003 (-0.006, 0.012) 0.554	**-0.029 (-0.043, -0.015) <0.001**	0.004 (-0.004, 0.013) 0.330	0.008 (-0.001, 0.016) 0.074	**-0.050 (-0.068, -0.031) <0.001**	-0.006 (-0.018, 0.005) 0.230	0.001 (-0.011, 0.013) 0.834
P for interaction	<0.001	0.030	0.001	<0.001	0.144	0.011	<0.001	0.001	<0.001
Stratified by age									
8–13	**-0.011 (-0.020, -0.002) 0.017**	-0.005 (-0.013, 0.004) 0.293	0.006 (-0.001, 0.014) 0.090	-0.008 (-0.017, 0.001) 0.090	-0.002 (-0.012, 0.007) 0.606	**0.011 (0.003, 0.019) 0.006**	**-0.020 (-0.030, -0.009) <0.001**	-0.008 (-0.018, 0.003) 0.158	0.006 (-0.004, 0.016) 0.218
14–19	**0.017 (0.008, 0.025) <0.001**	**0.015 (0.006, 0.023) <0.001**	**0.015 (0.007, 0.024) <0.001**	**0.027 (0.019, 0.036) <0.001**	**0.016 (0.008, 0.024) <0.001**	**0.019 (0.011, 0.026) <0.001**	-0.003 (-0.014, 0.007) 0.540	**0.012 (0.002, 0.023) 0.020**	**0.013 (0.002, 0.023) 0.016**
P for interaction	<0.001	0.001	0.002	<0.001	<0.001	<0.001	<0.001	0.003	0.019
Stratified by race									
Mexican American	0.014 (-0.003, 0.031) 0.096	-0.002 (-0.013, 0.009) 0.724	0.001 (-0.010, 0.011) 0.870	**0.023 (0.006, 0.040) 0.007**	0.004 (-0.007, 0.015) 0.480	0.008 (-0.002, 0.018) 0.120	0.009 (-0.011, 0.028) 0.399	0.004 (-0.009, 0.016) 0.573	0.006 (-0.007, 0.019) 0.333
Other Hispanic	0.016 (-0.010, 0.043) 0.233	0.001 (-0.016, 0.018) 0.913	**0.021 (0.006, 0.037) 0.008**	0.019 (-0.007, 0.045) 0.146	0.004 (-0.014, 0.021) 0.667	**0.026 (0.011, 0.041) <0.001**	0.020 (-0.013, 0.053) 0.238	0.008 (-0.012, 0.028) 0.429	**0.031 (0.011, 0.051) 0.003**
Non-Hispanic White	**0.034 (0.016, 0.052) <0.001**	**0.025 (0.014, 0.035) <0.001**	**0.026 (0.016, 0.035) <0.001**	**0.040 (0.023, 0.058) <0.001**	**0.029 (0.018, 0.039) <0.001**	**0.032 (0.022, 0.042) <0.001**	0.015 (-0.007, 0.037) 0.176	**0.017 (0.004, 0.030) 0.013**	**0.016 (0.003, 0.029) 0.017**
Non-Hispanic Black	**0.041 (0.021, 0.061) <0.001**	0.003 (-0.009, 0.015) 0.621	0.011 (-0.002, 0.023) 0.080	**0.039 (0.019, 0.059) <0.001**	-0.001 (-0.013, 0.012) 0.890	0.010 (-0.002, 0.021) 0.118	0.019 (-0.005, 0.044) 0.124	-0.011 (-0.027, 0.005) 0.174	0.007 (-0.009, 0.023) 0.410
Other Race	0.014 (-0.005, 0.032) 0.145	0.008 (-0.005, 0.021) 0.212	0.009 (-0.003, 0.021) 0.132	**0.027 (0.009, 0.045) 0.004**	**0.017 (0.004, 0.030) 0.012**	**0.019 (0.007, 0.031) 0.001**	-0.004 (-0.027, 0.018) 0.702	0.011 (-0.004, 0.026) 0.162	0.011 (-0.004, 0.026) 0.158
P for interaction	0.137	0.061	0.010	0.488	0.061	0.012	0.615	0.786	0.290

Model 1: No covariates were adjusted. Model 2: Age, gender, and race were adjusted. Model 3: Age, gender, race, PIR, BMI, serum vitamin D, serum total protein, serum calcium, serum phosphorus, blood urea nitrogen, cholesterol, serum uric acid, serum cotinine, and physical activity.

Abbreviation: BMD, bone mineral density. PIR, poverty income ratio. BMI, body mass index.

## Discussion

The present study explores the association between dietary copper intake and BMD in children and adolescents. We found a positive association between dietary copper intake and BMD in children and adolescents. For every 1mg/d increase in copper intake, the total BMD, subtotal BMD, and total spine BMD increased by 0.013 g/cm^2^, 0.020 g/cm^2^, 0.014 g/cm^2^. In addition, our results indicate that the positive relationship between dietary copper intake and BMD may vary across gender, age, and race. The effect is more pronounced in men, individuals aged 14–19, Non-Hispanic White, and Other Hispanic. Our study provides important evidence for the role of copper intake in bone health at the early stage of life.

The human body has approximately 50–80 mg of copper in dynamic balance, and two-thirds is in muscle and bone [22]. Cellular or animal studies have explored the effects of copper on bone metabolism [23]. In an early study, Li et al. [23] found that extracellular copper ions could inhibit osteoclast resorption. Moreover, copper could stimulate osteogenic differentiation of mesenchymal stem cells (MSCs) [24]. An animal study found low copper intake was associated with lower bone density in rats. In contrast, rats fed a diet with higher copper concentrations had higher bone strength [25]. Reduced enzyme activity due to copper deficiency may lead to connective tissue defects that can lead to bone problems [26]. Collagen is the principal protein in bone, giving it strength [27]. Jonas et al. [28] found that reduced mechanical strength of bones in rats due to a copper-deficient diet is associated with collagen loss.

Some epidemiological studies have explored the possible link between copper status and bone health [10,11,29,30]. In a recent study by Liu et al. [31], they found a negative association between serum copper and BMD in 910 U.S. adolescents aged 12 to 19. However, the small number of participants (only 910) may be a limitation of the study. Similar to our results, Wu et al. [32] revealed that serum copper was positively associated with BMD in children under three years after adjustments for potential confounding factors. However, research on the association between serum copper and bone health has mostly focused on the adult population. Mutlu et al. [33] found no statistically significant difference in serum copper between the control, osteopenic, and osteoporotic groups. However, in another study that included 728 Turkish postmenopausal women, Okyay et al. [30] found a significant inverse association between serum copper and lumbar and femur BMD. The authors attribute the results to the differences in study populations, methods, and others. Several studies also investigated the effects of copper supplementation on bone health but have also reached inconsistent conclusions [34,35]. In a randomized controlled trial (RCT) study of 224 postmenopausal women, Nielsen et al. [34] found that copper supplementation has no positive effect on BMD. However, in another study in the UK, Baker et al. [35] found that a high-copper diet (6.0 mg/d) could significantly reduce the blood markers of bone resorption in men, compared to a medium (1.6 mg/d) and low-copper diet (0.7 mg/d). Eaton-Evans et al. [36] evaluated the effects of 3 mg/d copper supplementation on bone health in 73 healthy women aged 45–56 years for two years. They found that copper supplementation could reduce bone loss in the lumbar spine. In total, most evidence suggested that copper status could positively impact bone health in adults. However, all these studies have been conducted in middle-aged or older adults. No studies have explored the association between copper intake and BMD in children and adolescents. Evidence showed that approximately 90% of PBM is achieved by the age of 18, usually called the "bone bank [37]." Berger et al. [38] found that women reached their hip PMB at 16–19 years, while men were at 20–30 years. PBM has been recognized as an independent risk factor for developing osteoporosis in later life, with a 10% increase in adolescence, delaying the onset of osteoporosis by 13 years and reducing the risk of fracture by 50% in later life [39,40]. Our study is the first to find a positive relationship between copper intake and BMD in children and adolescents. Therefore, adequate copper intake during childhood and adolescence may benefit bone health and could play a preventive role against osteoporosis in later life. However, more prospective studies are still needed to validate these results.

Our study indicated that the positive association between copper intake and BMD was stronger in individuals aged 14–19 years than at 8–13 years. Previous studies have shown that women reach their highest BMD growth rates at age 15 and men at age 17 [41]. Thus, the positive correlation of copper on BMD may be stronger in older children and adolescents because BMD is more likely to be affected during these periods. In addition, we found that the positive relationship between copper intake and BMD was more pronounced in non-Hispanic whites and Other Hispanic. There are racial differences in the development of osteoporosis. Whites are the race with the highest osteoporosis prevalence [42]. Genetic differences between races may partly account for these differences, as studies suggest that 50% to 85% of PBM is genetically determined [43]. Thus, the effect of copper intake on BMD across races may be attributed to genetic differences. Interestingly, we found that the positive relationship between copper intake and BMD was more pronounced in men, not women. One explanation is that adolescent men consume more copper than adolescent women. In our study, daily copper intake in our study was 1.00 ± 0.43 and 0.86 ± 0.34 mg/d for adolescent men and women, respectively. However, few studies have explored gender differences in the effects of copper on bone health. Further studies are needed to investigate the different effects of copper intake and BMD in different sexes and their underlying mechanisms.

Our study has some advantages. First, we are the first to evaluate the association between copper intake and BMD in children and adolescents. Second, we used a large sample and performed sample weighting, and the results can be generalized to the general U.S. population. Third, we adjusted a large range of confounding variables to ensure the reliability of the results. Our study also has some limitations. First, due to the nature of cross-sectional studies, we cannot infer causality. Further prospective studies are needed to confirm our results. Second, our study did not include children under 8 years of age because NHANE did not include BMD data for these children. Third, copper intake in our study was assessed based on dietary recall and may be subject to recall bias.

## Conclusions

Our study suggests that copper intake is positively associated with BMD in U.S. children and adolescents. The study emphasizes the role of copper intake on bone health in the early stages of life. However, more investigations are needed to verify our findings and their underlying mechanisms.

## Supporting information

S1 Appendix Detailed information on participants in this study. (XLSX)
